# Effects of caffeine intake on pupillary parameters in humans: a systematic review and meta-analysis

**DOI:** 10.1186/s12993-024-00245-y

**Published:** 2024-08-05

**Authors:** Elias Vincent Hartmann, Carolin Franziska Reichert, Manuel Spitschan

**Affiliations:** 1 Centre for Chronobiology, University Psychiatric Clinics Basel (UPK), Basel, Switzerland; 2 Transfaculty Research Platform Molecular and Cognitive Neurosciences, University of Basel, Basel, Switzerland; 3 Max Planck Institute for Biological Cybernetics, Translational Sensory & Circadian Neuroscience, Tübingen, Germany; 4 TUM School of Medicine & Health, Technical University of Munich, Munich, Germany; 5 TUM Institute for Advanced Study (TUM-IAS), Technical University of Munich, Garching, Germany

**Keywords:** Caffeine, Psychophysiology, Pupil, Pupillometry

## Abstract

Caffeine is a widely used drug that broadly affects human cognition and brain function. Caffeine acts as an antagonist to the adenosine receptors in the brain. Previous anecdotal reports have also linked caffeine intake with changes in pupil diameter. By modifying the retinal irradiance, pupil diameter modulates all ocular light exposure relevant for visual (i.e., perception, detection and discrimination of visual stimuli) and non-visual (i.e., circadian) functions. To date, the extent of the influence of caffeine on pupillary outcomes, including pupil diameter, has not been examined in a systematic review. We implemented a systematic review laid out in a pre-registered protocol following PRISMA-P guidelines. We only included original research articles written in English reporting studies with human participants, in which caffeine was administered, and pupil diameter was measured using objective methods. Using broad search strategies, we consulted various databases (PsycINFO, Medline, Embase, Cochrane Library, bioRxiv and medRxiv) and used the Covidence platform to screen, review and extract data from studies. After importing studies identified through database search (n = 517 imported, n = 46 duplicates), we screened the title and abstracts (n = 471), finding 14 studies meeting our eligibility criteria. After full-text review, we excluded seven studies, leaving only a very modest number of included studies (n = 7). Extraction of information revealed that the existing literature on the effect of caffeine on pupil parameters is very heterogeneous, differing in pupil assessment methods, time of day of caffeine administration, dose, and protocol timing and design. The evidence available in the literature does not provide consistent results but studies rated as valid by quality assessment suggest a small effect of caffeine on pupil parameters. We summarize the numeric results as both differences in absolute pupil diameter and in terms of effect sizes. More studies are needed using modern pupil assessment methods, robust study design, and caffeine dose–response methodology.

## Introduction

Caffeine is a common stimulant consumed by the majority of adults all over the world [1, 2]. The primary mechanism of caffeine action in the human body can be assigned to its ability of adenosine antagonism [3, 4]. Caffeine has been shown to impact circadian rhythms and sleep behaviour in human and animal studies [5–8]. Previous research has shown that caffeine may potentiate non-image-forming effects of light in animals and humans, for instance, on the timing of rest-activity cycles or on melatonin secretion [9–13]. Therefore, caffeine may represent a tool to enhance light effects on the biological clock.

The pupil acts as a gate regulating the amount of light entering the eye, thereby modifying light information at the earliest stage. The pupil diameter, which undergoes changes with age [14–19] and is linked to other ocular functions through the near triad involving vergence and accommodation [20], adjusts to environmental light through the pupillary light reflex (PLR). The PLR is controlled by the sympathetic and parasympathetic nervous system [21–23] and is therefore influenced by a range of inputs, including mental load, mood and alertness [19]. By stimulating the release of catecholamines, caffeine can increase activity in this autonomic nervous system. Acute caffeine and caffeine metabolites can promote the synthesis, release and turnover of central noradrenaline (NE) [24–27], e.g., in the locus coreuleus (LC) [24] and the brainstem [25]. LC-NE activity in turn increases pupil size and affects its response to light [22, 23, 28]. Therefore, it is reasonable that the stimulant properties of caffeine could potentially modulate pupil regulation.

In the eye, the intrinsically photosensitive ganglion cells (ipRGCs) in the human retina represent the “front end” of the circadian system. The ipRGCs express the short-wave sensitive photopigment melanopsin [29–31]. Selective activation of melanopsin modifies retinal illumination by reducing pupil diameter [32]. Previous research on retinal ganglion cells in the rat suggests that adenosine agonists reduce light-evoked spiking of ipRGCs via the adenosine A1 receptor [33]. Further on, adenosine antagonists reverse this effect. As an unspecific antagonist at adenosine receptors, caffeine may work similarly and alter light-induced pupil responses by blocking A1 receptors at ipRGCs.

While caffeine is known to have widespread effects on brain function, the extent to which it modifies pupil diameter is currently unknown. A potential influence on pupil diameter could explain why caffeine has the potential to enhance light effects on the circadian system. In this systematic review, we focus on whether caffeine intake affects pupil control in humans, closing a critical gap in the literature.

## Methods

### Pre-registration and reporting guidelines

The protocol for the systematic review in hand was registered on the Open Science Framework (OSF; https://osf.io/a5ymd/) [34]. We followed the PRISMA-P guidelines (2020) [35] for systematic reviews.

### Selection criteria

We conducted a systematic literature search to evaluate the effects of caffeine intake on pupil control in humans. Only primary literature in the English language was included, involving both healthy and clinical populations. No date range was applied for inclusion. Articles published as reviews, editorials, letters to the editor, opinion papers or book chapters were excluded.

Additionally, we applied the following selection criteria:

- Caffeine had to be administered in the study. The form of administration was optional and could include coffee, tea, energy drinks, capsules, pills and others.
- The study design had to include at least one control condition (e.g., baseline measurement or comparison with placebo).
- Pupil diameter had to be measured using objective methods (incl. photographic, videographic and ruler methods).

### Information sources

To obtain a complete overview of the subject, we searched various libraries. A total of six platforms were consulted for the literature search. Literature research was split up into two parts. On 19 April 2021, Medline, Embase, and PsycINFO were searched for relevant papers. They were then imported into Covidence on 20 April 2021. On 28 April 2021, medRxiv and bioRxiv were searched manually through their respective web interfaces (https://medrxiv.org/ and https://biorxiv.org/) and likewise imported into Covidence. All references were uploaded in RIS format. The search strategy used is further elaborated in the following section. To include full-text articles, we examined the reference lists and screen-cited papers for relevance using the titles of the cited articles.

### Search strategy

Our search strategy involved a search of several databases in order to make sure to include all relevant studies. A total of six databases were searched, providing literature mainly on medicine, biology and psychology.

We searched PsychINFO, Medline and Embase using the search terms “(caffeine and pupil*).af.”. At which “.af” stands for “all fields” and means that the searched terms must not only be found over the title of papers but can be found in the abstract, main text or else. The other databases were queried with the strategies shown in Table 1.

**Table 1:** The search strategy used for literature research

• PsychINFO: (caffeine and pupil*).af • Medline: (caffeine and pupil*).af o In addition, we implemented the following subject heading-based strategy: • 1 Pupil/ • 2 (pupil* adj3 (size or enlarge* or large* or dilat* or small or constrict* or contract*)).ti,ab,kw • 3 pupil*.ti • 4 1 or 2 or 3 • 5 Caffeine/23720 • 6 coffee/or energy drinks/or tea/ • 7 (caffeine* or coffee or tea or energy drink?).ti,ab,kw • 8 5 or 6 or 7 • 9 4 and 8 • Embase: (caffeine and pupil*).af • Cochrane Library: caffeine pupil* • bioRxiv: https://www.biorxiv.org/search/caffeine%252Band%252Bpupil%252A • medRxiv: https://www.medrxiv.org/search/caffeine%252Band%252Bpupil%252A	

### Processing

We used Covidence (Veritas Health Innovation, Melbourne, Australia; available at https://www.covidence.org/) to streamline literature research processes. PsychINFO, Medline, and Embase search results were exported to Covidence in RIS format. Cochrane, bioRxiv, and medRxiv datasets were similarly imported as RIS files. Covidence automatically screened for duplicates, normalizing titles by ignoring special characters and punctuation. It then checked for matching titles, year digits, volume, and authors, excluding duplicates. Two raters independently screened titles and abstracts, obtaining full texts through Oxford and Basel library accounts. Two scorers reviewed full texts, resolving conflicts at a project meeting.

### Data extraction

Data were systematically organised and divided into five subsections:

- Article Information: a list of validated identifiers for each article, e.g. title and Digital object identifier (DOI).
- Study design: a quick overview of the experimental design.
- Sample details: the most relevant descriptions of the participants included in each study.
- Caffeine details: summarising facts about the active manipulation variables of the studies.
- Pupil diameter measurement details: information about the collection of the dependent variable in the studies.

This plan was then used for all the studies found by the search strategy. The extracted data were then added to Covidence as a Data extraction template. Data were then added manually and by two raters, working independently, who picked out the relevant terms found in the papers. Missing information was classified as: none, not/none reported (n.r.) or other, but entered in the table as data. Table 2 shows all the variables and data outcomes extracted from the present studies.

**Table 2:** Data extraction variables

Article information	Study design	Sample details	Caffeine details	Pupil size measurement details
Title of paper	Description of design	Sample size	Self-reported daily caffeine intake	Design
Authors of paper	Type of study	Number of study groups	Prior caffeine intake control reported?	Pupil size measurement method
DOI	Blinding method	If patient group included, describe condition	Abstinence	Pupil size measurement method performance characteristics
PubMed ID	Within- or between-subjects design	If patient group included, describe diagnostic method	Abstinence duration	Stimulus characteristics
Journal	Time-of-day of study controlled?	Number of female participants per group	Caffeine concentration measured	Main pupil size measure
Publication year	Time-of-day control method	Number of male participants per group	Any further details on caffeine intake control method	General statistical strategy
Funding reported?	Measured outcomes	Mean or median age per group	Caffeine dosing	Report of pupil size values
Funders		Age range per group	Caffeine dose	Report of effect size
		Inclusion criteria per group	Method of administration	Pupil size values
		Exclusion criteria per group	Administration details	Effect size values
		Descriptive variables of sample per group	Type of control	*p* values of any statistical tests
			Description of placebo, if included	

### Quality assessment

To critically assess studies for methodological problems, we used the quality assessment template included in Covidence as the default: the Cochrane risk of bias (RoB) template for randomised controlled trials (RCT) [36]. The investigators deliberately chose this high standard of RCTs, even though it was not an inclusion criterion for the studies to be RCTs. One rater (EH) screened studies for RoB using the Cochrane Risk of Bias tool 2.0 for RCTs [37]. Each question had to be answered with an assignment of either “Low”, “High”, or “Unclear”.

The RoB tool consists of questions on seven domains of potential biases:

- Random sequence generation (Should the randomisation generate comparable groups?)
- Allocation concealment (Could allocation have been foreseen?)
- Blinding of personnel and participants (Could knowledge of the allocated intervention bias participants’ performance or personnel during the study?)
- Blinding of outcome assessment (Could knowledge of the outcome assessor about the allocated intervention group bias the evaluation?)
- Incomplete outcome data (Was the handling of missing data adequate and should not produce bias?)
- Selective reporting (Is there a possibility of selective outcome reporting?)
- Other sources of bias (Are there any other concerns that could produce bias?)

### Synthesis

We were not able to perform quantitative evaluations because the seven included studies different marjorly in study design, caffeine administration, and pupil measurement methods. Therefore, we elected to conduct a qualitative review. A table visualising the data extracted by the above method is provided to overview relevant variables in each study. Missing data points are reported as “none”, “not/none reported (n.r.)”, or “other”. Further, no transformations of outcome measurements were performed, nor was any statistical software used besides Excel.

## Results

### Summary of included and analysed studies

**Fig. 1:**
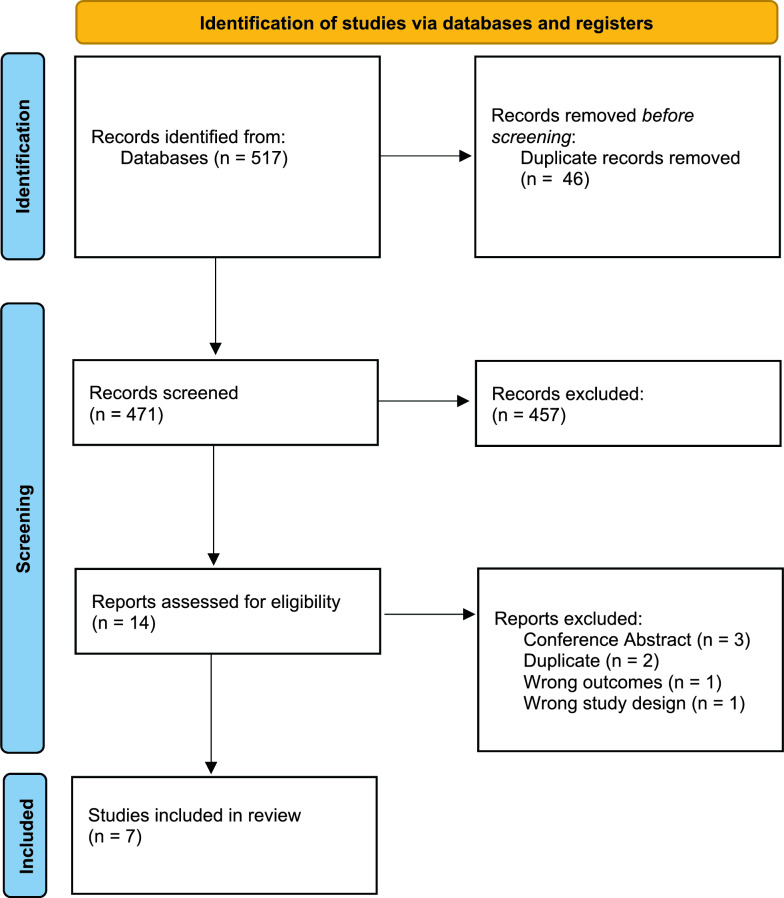
PRISMA flowchart of the literature research process

Figure 1 summarises the selection process of included studies. Our initial search strategy yielded 517 studies. The tool searching for duplicates in Covidence identified and subsequently excluded 46 duplicates. After duplicates were removed, 471 studies remained. The remaining studies made it to the next step, based on screening of titles and abstracts. Two independent raters (E.H. and M.S.) screened the studies independently. Afterwards, conflicts were discussed in a Zoom meeting of three (with C.F.R.) and C.F.R. resolved the conflicts. A total of 457 studies were excluded in this step as they were irrelevant or did not meet the inclusion criteria. The large majority of studies excluded in this step were due to incidental match through the search strategy.

Three references had to be excluded because they turned out to be conference abstracts. As mentioned before, our inclusion criteria stipulated that we would only include primary research. Articles published as a review, editorial, letter to the editor, opinion paper or book chapter were thus excluded.

A total of seven studies were included in the systematic review. The studies all consist of a small study sample of between 5 and 60 participants each. In all studies, caffeine was administered as part of the protocol. All but one study are constructed in a within-subject design. Still, there were three different forms of administration. Since coffee consists of numerous chemical compounds, potential effects cannot be attributed to caffeine alone as long as it is not compared to caffeine-free coffee as a placebo. However, neither included study involving coffee included a control condition. The studies are grouped by the method of caffeine administration in Table 3.

**Table 3:** Overview of the included studies

Study	Study design	Blinding method	Within- or between-subjects design	Sample size	Abstinence duration (in hours)	Method of administration	Caffeine dosing	Type of control	Results
Redondo et al. [38]	Crossover study	Double-blind	Within-subjects	22	12	Oral (capsule)	4 mg/kg	Baseline measurement and Placebo	Caffeine significantly increased pupil size compared to placebo
Lanini et al. [39]	Randomised, placebo-controlled, independent-group design study	Double-blind	Between-subjects	60	12	Oral (capsule)	Habitual breakfast dose of caffeine (Ranged between 25 and 300 mg)	Baseline measurement and Placebo	Baseline difference in pupil size between groups; no interaction with time
Abokyi et al. [40]	Double-blind, placebo-controlled, randomised crossover experimental design	Double-blind	Within-subjects	50	168	Oral (drink)	250 mg	Baseline measurement and Placebo	Pupil diameter increased from 3.4 (± 0.4 mm) at baseline to 4.5 (± 0.72 mm) at 90 min
Krueger et al. [41]	Placebo-controlled, within-subject design	Simple-blind	Within-subjects	5	24	Oral (drink)	350 mg caffeine/75 kg bodyweight	Baseline measurement and Placebo	No influence of caffeine on pupil dilation caused by pain
Wilhelm et al. [44]	Open study design	No blinding	Within-subjects	20	9	Oral (drink)	Caffeine content of 86.85 mg per cup (about 1–1.5 mg/kg)	Baseline measurement	Caffeine caused a reduction of pupillary unrest index (PUI)
Bardak et al. [42]	Non-randomised design	No blinding	Within-subjects	30	12	Oral (drink)	57 mg	Baseline measurement	tendency of increased pupil size in the caffeine group
Nicholson et al. [43]	Randomised controlled trial (RCT)	Double-blind	Within-subjects	6	32	Oral (ingestion)	300 mg	Baseline measurement and placebo	No significant changes in pupil size

### Detailed description of included and analysed studies

All included and analysed studies are summarised in Table 3. Redondo et al. conducted a placebo-controlled, double-blind, balanced, crossover study [38]. The study sample contained 22 participants (male and female) and employed a within-subject design. Before the experimental session, participants had to abstain from caffeine for 12 h. Caffeine was administered in capsules at the same time of the day at 4 mg/kg body weight doses. Pupil measurement was performed with a binocular autorefractometer. Pupil dilation was found 30 min after caffeine ingestion and was statistically significant. This study was found to have the lowest risk of bias (RoB) of all included studies in this review.

Lanini et al. performed a randomised, placebo-controlled, double-blind, independent-group-design study [39]. The between-subject study was carried out on 60 men. Subjects abstained from caffeine for 12 h prior to the beginning of the study. The administered capsule contained the amount of caffeine corresponding to each participant’s habitual breakfast caffeine dose. In addition, a standardised meal was given in the form of cereal bars. Pupil diameter measurements were performed with a video-based infrared camera. The caffeine and non-caffeine-treated groups differed as to pupil diameter, but this reflected a baseline group difference that was not altered significantly by treatment over time.

The study of Abokyi et al. [40] was executed as a double-blind, placebo-controlled, randomised crossover experimental study with a within-subject design. The experimental sample consisted of 50 study participants (male and female). In advance of the study, participants were asked to abstain from caffeine for a week (168 h). The caffeine dose was 250 mg and was served in a lemon drink. Pupil diameter was measured by clinicians using a ruler. Repeated measures subjected to one-way ANOVA showed a significant increase in pupil diameter with time after subjects consumed caffeine.

In another placebo-controlled study with a within-subject design by Krueger et al. [41], the sample consisted of five male participants. A 24-h period of caffeine abstinence preceded the experimental sessions. The caffeine dosing was set to 350 mg/75 kg body weight. It was administered in a bitter-sweet drink. Pupil measurements were performed using an infrared-light and video-based technique. The study did not find any effects of caffeine on pupil diameter. However, the study mainly focused on caffeine’s potential analgesic effect.

Bardak et al. [42] conducted a non-randomised experimental study investigating the effect of caffeine compared to the baseline measurement. Thirty healthy occasional coffee drinkers were included as participants. The sample involved 14 male and 16 female subjects. The participants were instructed not to drink any caffeinated drinks or take any medication for at least 12 h before the study. Caffeine was served in a cup of Turkish coffee containing 57 mg of caffeine. Pupil diameter was measured using an irx3 wavefront aberrometer (Imagine Eyes, Orsay, France). Pupil diameter seemed to increase a bit in the caffeine group. However, it did not reach any significant changes over time.

Nicholson et al. [43] carried out a randomised, within-subject, placebo-controlled trial, including six female participants. Abstinence was enforced for 32 h prior to the laboratory session. The caffeine dose was 300 mg, administered orally. A television pupillometer measured pupil diameter with an infrared-sensitive camera. After any treatment, there were no changes in maximum, minimum, mean, or final pupil diameter in their study.

The study by Wilhelm et al. [44] did not focus on pupil diameter but instead on pupillary oscillations and represents a special case. The focus was not on characterising differences in pupil diameter, but on the application of pupillary oscillations in detecting sleepiness. Due to the use of caffeine, it is included here. The design was a non-randomised open design did not involve blinding in contrast to the other studies. The within-subject sample consisted of 20 participants (male and female). The duration of abstinence prior to the study was 9 h. Caffeine was administrated in the form of coffee, served in cups that contained 86.85 mg of caffeine per cup (about 1–1.5 mg/kg). Pupil diameter was measured with a video-based device and quantified further in the pupillary unrest index (PUI) [45]. Caffeine caused a reduction in the PUI, with a maximum effect 1.25 h after consumption.

### Summary of risk of bias

**Fig. 2:**
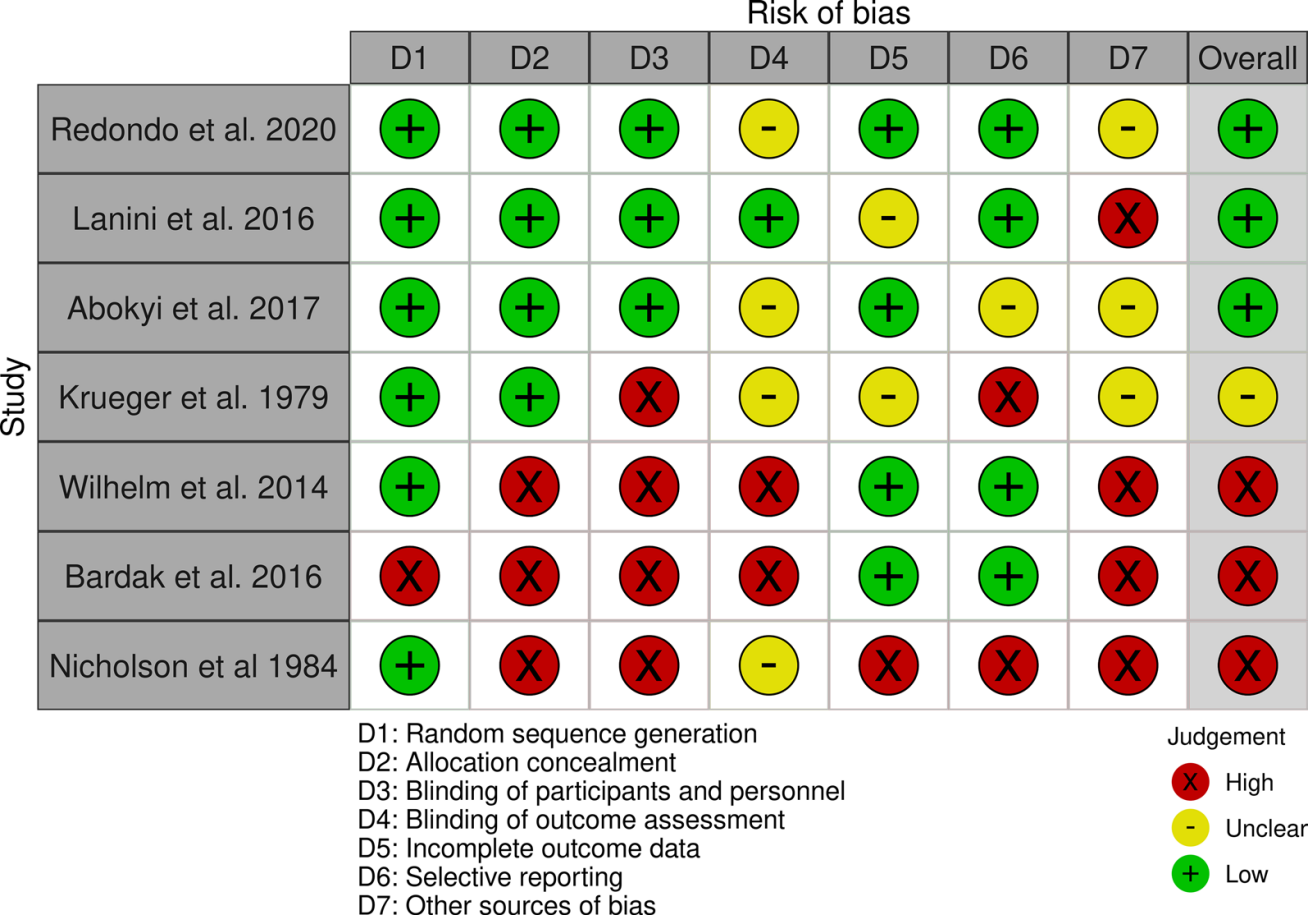
Traffic-light plot to Cochrane risk of bias tool

The RoB traffic-light plot (Fig. 2) visualises the assessments for each RoB question. It shows the diversity in methodological rigor of the included studies. The wide range of 40 years between publication dates of the youngest and oldest included studies stands out regarding methodological quality. The distribution of favourable and unfavourable methods is rather even. Three studies each are rated to have a total low or high overall bias. Most studies were classified to fulfil the “Random sequence generation” criterion. On the other side, blinding of participants and personnel had to be met four times with a “high risk of bias”. Lack of allocation concealment has also been identified in three studies as a risk for bias. It is important to note that the criteria for RoB assessment used here are relatively conservative. Indeed, automated measurement techniques (e.g. used in some studies, [44]) could have resulted in an actual bias.

### Summary of effects and directionalities

**Fig. 3:**
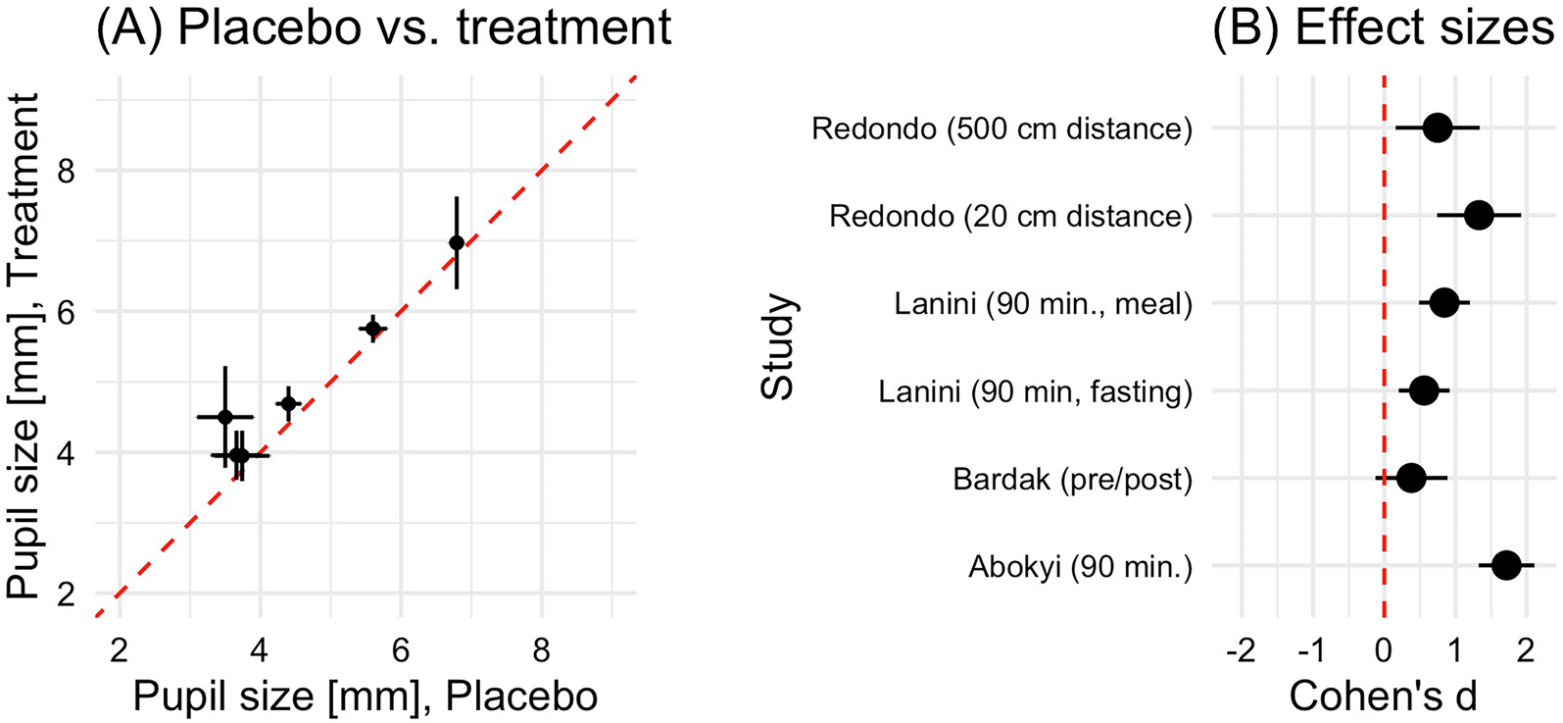
Quantitative summary of results

The studies on hand do not provide consistent results. Some studies seem to find an effect of caffeine on pupil diameter in the form of a dilation, visualised in Table 3 and Fig. 3 [38, 40]. The study by Redondo et al. [38] finds a significant main effect of caffeine consumption on pupil diameter (F1, 21 = 7.812, p = 0.011, η2 = 0.271) 30 min after capsule ingestion, compared to placebo. The publication of Abokyi et al. [40] provides data on pupil dilation in the caffeine group compared to a placebo group as well. The baseline pupil measurement of the caffeine group revealed a mean pupil diameter of 3.4 mm [± 0.4 mm Standard deviation (SD)]. 90 min after caffeine consumption, the mean pupil diameter reached 4.5 mm (± 0.72 mm SD). By this, the caffeine intake group shows significantly greater effects than placebo at time points 30, 60, and 90 min (p < 0.001). The study of Lanini et al. [39] finds a significant difference between the caffeine and non-caffeine groups. However, this difference did not interact with time and returned to a baseline group difference. The study by Wilhelm et al. [44] found a clear effect on the pupillary unrest index.

The articles by Krueger et al. [41], Bardak et al. [42] and Nicholson et al. [43] could not provide data supporting the thesis of pupil dilation after caffeine consumption whatsoever. Therefore, most of the included studies cannot support the thesis of pupil dilation due to acute caffeine intake. In the study of Bardak et al. [42] a very small caffeine dose (57 mg) was chosen. Lanini et al.’s [39] study used participants’ habitual breakfast dose, which ranged between 25 and 300 mg. First, 25 mg of caffeine appears to be a very small caffeine dose. A regular cup of coffee of 125 ml contains about 100 mg of caffeine in comparison. About 90% of adults report an average daily caffeine intake of 227 mg [1, 46]. However, these studies did not specifically set out to test the effect of caffeine. Only two to three studies meet generally accepted modern methodological study criteria for and provide reliable data on pupil diameter. These studies seem to find a small effect of acute caffeine intake in humans on pupil size and other pupil parameters.

## Discussion

This review investigated how acute caffeine intake may induce pupil dilation in humans. The few studies included insufficient data available to draw clear conclusions to the present research question. The methodological tools of each study differ from those of the other studies included in this paper. It is not possible to use quantitative methods with the data on hand. The range of different caffeine doses used in these studies makes it impossible to find consistent effects. A small caffeine dose, combined with the disregarding of adequate abstinence durations and early administration times, can lead to negligible effects on pupil control.

### Limitations of this systematic review

We chose to include papers in the English language only in this systematic review. The predominance of English-language journals with a high impact factor might be associated with a higher probability of studies publishing positive results in specific journals. We may therefore have missed relevant studies written in a language other than English. We also chose not to include grey literature in the current systematic review.

In some of the included studies, pupil diameter is not the primary object of investigation [39, 41, 43, 44]. These studies contain confounding variables that reduce or even negate their validity for the research question at hand. Those studies [38, 40] that are methodologically sound and focus on pupil diameter also find a small effect of caffeine on pupil diameter.

Some of the included studies come with methodological shortcomings. This threatens explanatory power. Two studies [41, 43] suffer from a small sample size of fewer than seven participants. Two other studies have no blinding at all in their study design. The study by Krueger et al. [41] only provides single blinding of study participants, which does not meet contemporary standards. Placebo is included in all but two studies [42, 44], in which comparison is only to a baseline measurement. These two studies also stand out because of their study design in general. The study by Wilhelm et al. [44] used an open study design, and Bardak et al. [42] used a non-randomised study design.

Overall, there were inconsistencies in the sex of the sample. While some studies had rather balanced samples [44], the publications by Lanini et al. [39] and Krueger et al. [41] included men only, while the study sample by Nicholson et al. consists only of women.

The included studies were highly variable and did not employ standardized techniques discussed by [47]. Differences in background illuminance, stimulus conditions (including wavelength, duration, dark adaptation), data collection techniques, and preprocessing and analysis strategies add to the heterogeneity in the literature.

### A roadmap for research in the future

*The need for well-controlled trials.* It would be crucial to perform large placebo-controlled, double-blind RCTs to generate valid results on the impact of caffeine on pupil diameter and pupil-derived parameters. In a next step, studies should disentangle the relation of caffeine-dependent effects on pupil diameter, such as autonomic nervous system activity or light-induced ipRGC activity. However, we recognize the importance of contextualizing these results within real-world scenarios, where factors such as individual sensitivity to caffeine consumption and its timing may play a significant role.

*Focus on individual differences.* Future studies should also investigate contextual influences and inter-individual differences, such as individual differences in adenosine or adenosine receptor sensitivity, which may help explain the absence of effects in some individuals and predict caffeine-dependent enhancement of the impact of light on neuroendocrine and circadian physiology.

*Efficacy vs. effectiveness.* The question of efficacy versus effectiveness remains. A rather ideal setting for determining the effects of caffeine is in caffeine-naïve or abstinent individuals not exposed to influences on caffeine metabolism (such as smoking or hormonal contraceptives) and who subsequently consume more than 3 mg/bodyweight caffeine in the evening—after several hours of wakefulness. Of course, these conditions do not necessarily reflect the habits of most people. Studies tend to select a particular sample of participants who do not necessarily represent the general public. Especially in studies involving caffeine and caffeine abstinence, a deviation from common consumption patterns in the real world can be expected.

*Administration parameters.* Previous studies have found some characteristics of caffeine administration that seem to have a significant influence on potential effects. Firstly, the previous time spent awake before caffeine administration is crucial. The effects of caffeine administered in the morning right after awakening have been shown to differ from those produced by caffeine in the evening due to differences in adenosine occurrence [8, 48, 49]. One explanation for this is that the effect of caffeine is dependent on adenosine levels, which increase during extended time spent awake [50].

*Controlling for caffeine.* The previous caffeine consumption of participants before the study must be kept in mind. Tolerance to caffeine may develop within several days and dampens the stimulating effect of the substance. This can be reduced by determining the duration of abstinence prior to and throughout the experiment. Ideally, compliance with the regimen should be controlled by saliva or blood sampling. The specific duration to achieve a caffeine-sensitive or "clean" state, however, is still unknown and may differ inter-individually. Abstinence durations of 12–24 h chosen in the studies discussed seem to be too short, as withdrawal symptoms peak between 20 and 51 h and indicate an ongoing process of adaption in the adenosine system [51]. During daily consumption, caffeine metabolism may not complete within 24 h and active degradation products are present [52]. Paraxanthine is considered the primary metabolite of caffeine and has similar effects on the human body, for instance, the blockade of adenosine receptors [46]. To capture the full potential of a single caffeine dose, caffeine-naïve participants would show the most apparent effects. The administration of caffeine should also be monitored restrictively in future studies. It is imperative that diet, medication intake, habituation and placebo effects are monitored and maintained constant between patients of a study. Caffeine-naïve participants should be included as a form of control to estimate the influence of habitual caffeine intake prior to the performed study. Ideally, there should also be further checks of compliance of caffeine withdrawal.

*Standardised and harmonized conducting and reporting of pupil studies*. Pupil measurements should be conducted and reported consistently and precisely. Recently published guidelines for pupillary research methodology represent an excellent starting point for future research [47, 53]. The recommendations by Kelbsch and colleagues [47] are comprehensive and recommend data collection and reporting standards for a series of common pupil measurement paradigms, including measurements of the afferent and efferent pupil control pathways. Beyond measuring pupil diameter, it will be critical to measure other pupil parameters, including spontaneous pupillary oscillations [45], which have been found to be sensitive to caffeine [44], under a wide range of parametric stimulus variations and using standard processing tools.

## Conclusion

In conclusion, the systematic review aimed to explore the potential impact of acute caffeine intake on pupil control in humans, filling a crucial gap in the existing literature. The findings from the included studies exhibit a lack of consistency and conclusive evidence regarding the effects of caffeine on pupil diameter. Methodological variations, small sample sizes, and confounding factors limit the available data. While some studies suggest a possible association between caffeine intake and pupil dilation, the results are not robust enough to draw firm conclusions.

The diverse methodologies employed in the studies, such as varying caffeine doses, different measurement techniques, extracted parameters and analytic strategies, and dissimilar participant characteristics, hinder direct comparisons and generalisations. Moreover, several studies lacked appropriate blinding or placebo controls, raising concerns about potential biases. The included studies overall highlight the need for more rigorous research designs that adhere to current standards.

To address these limitations and provide more conclusive insights, future research should focus on well-designed randomised controlled trials with larger sample sizes, using consistent caffeine doses and administration methods. Attention should be given to minimising confounding variables, such as prior caffeine consumption and withdrawal effects, to isolate better the true effects of acute caffeine intake on pupil control. Incorporating modern pupillometric techniques and addressing inter-individual variations in caffeine sensitivity will further enhance the validity of the findings.

In summary, while the current systematic review highlights the potential for caffeine to influence pupil diameter, it underscores the need for high-quality studies to establish a clearer understanding of this relationship. The findings emphasise the importance of refining experimental protocols and adhering to rigorous methodological standards to pave the way for more definitive conclusions regarding the impact of caffeine on pupil control and its potential implications for circadian rhythms and light responses.

## Data Availability

All data and materials can be downloaded under a Creative Commons license (CC-BY-NC-ND) from our GitHub repository: https://github.com/tscnlab/.

## Abbreviations

ipRGCs: Intrinsically photosensitive retinal ganglion cells
LC-NE: Locus coeruleus-norepinephrine
PLR: Pupillary light reflex
PUI: Pupillary unrest index
RCT: Randomised controlled trial
RoB: Risk of bias
SCN: Suprachiasmatic nucleus
SD: Standard deviation
